# Same species, different risks: predation shapes hormonal profiles in a breeding sea duck, the common eider

**DOI:** 10.1093/conphys/coag036

**Published:** 2026-06-24

**Authors:** Bertille Mohring, Farshad S Vakili, Frédéric Angelier, Kim Jaatinen, Suvi Ruuskanen, Olivier Chastel, Charline Parenteau, Bin-Yan Hsu, Titiksha Peetumber, Céline Arzel, Markus Öst

**Affiliations:** School of Environmental Sciences, University of Liverpool, 4 Brownlow Hill, Liverpool L69 3GJ, UK; Environmental and Marine Biology, Åbo Akademi University, Henriksgatan 2, 20500 Turku, Finland; Centre d’Etudes Biologiques de Chizé, UMR 7372 CNRS—La Rochelle Université, 405 Rte de Prissé la Charrière, 79360 Villiers-en-Bois, France; Department of Biology, University of Turku, Vesilinnantie 5, 20014 Turku, Finland; Centre d’Etudes Biologiques de Chizé, UMR 7372 CNRS—La Rochelle Université, 405 Rte de Prissé la Charrière, 79360 Villiers-en-Bois, France; Nature Solutions, Finnish Environment Institute, Latokartanonkaari 11, 00790 Helsinki, Finland; Department of Biological and Environmental Sciences, University of Jyväskylä, Survontie 9, 40500 Jyväskylä, Finland; Centre d’Etudes Biologiques de Chizé, UMR 7372 CNRS—La Rochelle Université, 405 Rte de Prissé la Charrière, 79360 Villiers-en-Bois, France; Centre d’Etudes Biologiques de Chizé, UMR 7372 CNRS—La Rochelle Université, 405 Rte de Prissé la Charrière, 79360 Villiers-en-Bois, France; Department of Life Science, Tunghai University, No. 1727, Sec. 4, Taiwan Boulevard, Xitun District, Taichung City 407224, Taiwan R.O.C; Department of Biology, University of Turku, Vesilinnantie 5, 20014 Turku, Finland; Department of Biology, University of Turku, Vesilinnantie 5, 20014 Turku, Finland; OMPO, 59 rue Ampère, 75017 Paris, France; Environmental and Marine Biology, Åbo Akademi University, Henriksgatan 2, 20500 Turku, Finland; Department of Bioeconomy, Novia University of Applied Sciences, Raseborgsvägen 9, 10600 Ekenäs, Finland

**Keywords:** Corticosterone, energy expenditure, human disturbance, parental investment, prolactin, scarecrow effect*, Somateria mollissima*; stress response, thyroid hormones, tourism

## Abstract

Prey are predicted to respond to humans as they would to predators. Hence, human disturbance can elicit costly physiological and behavioural antipredator responses. However, human presence can also shelter prey from human-averse predators (the human ‘scarecrow effect’). Whether this ‘scarecrow effect’ reduces perceived predation risk and promotes prey reproductive investment, as predicted by life-history theory, remains unclear. To test this, we monitored complementary hormonal markers in two geographically proximate colonies of common eiders *Somateria mollissima* breeding under contrasting conditions: one exposed to low human disturbance but high predation risk, and the other to high human disturbance (intense tourism) shielding birds from predators (i.e. low predation risk). We measured proxies of energy expenditure [baseline corticosterone (CORT)] and thyroid hormone levels [triiodothyronine (T3) and thyroxine (T4)], acute stress response (stress-induced CORT levels) and parental effort [baseline prolactin (PRL) levels]. Hormonal profiles differed markedly between colonies. Eiders in the low-disturbance/high-predation colony displayed higher baseline CORT and T3 levels than those in the high-disturbance/low-predation one, suggesting higher energy expenditure—possibly reflecting a higher energetic cost of incubating under elevated predation pressure. Stress-induced CORT levels were similar across colonies, suggesting that eiders maintain sensitivity to acute stressors despite a human ‘scarecrow effect.’ Supporting the hypothesis of increased parental effort under reduced perceived predation risk, incubating females displayed higher baseline PRL levels in the high-disturbance/low-predation colony. Altogether, these results are consistent with a human ‘scarecrow effect’ that reduces perceived predation risk without diminishing sensitivity to acute stressors, and that is associated with increased reproductive investment. Our research highlights the value of integrating multiple hormonal markers to understand wildlife responses to human disturbance and predation risk.

## Abbreviations

BMR: basal metabolic rate
CORT: corticosterone
ELHS: emergency life-history stage
GC: glucocorticoids
HPA: hypothalamo–pituitary–adrenal
LM: linear model
PRL: prolactin
T3: triiodothyronine
T4: thyroxine
TH: thyroid hormones

## Introduction

Predators shape prey population dynamics and phenotypic traits through both direct (consumptive) and indirect (non-consumptive) effects (Lima, 1998; Zanette and Clinchy, 2017). The mere risk induced by the presence of a predator in the environment may create a landscape of fear (Laundré *et al.*, 2001, 2010), associated with adjustment of prey behaviour, physiology, morphology or life history (Lima and Dill, 1990; Sheriff *et al.*, 2009; Bourdeau and Johansson, 2012; Kvile *et al.*, 2021). As prey are predicted to respond to humans as they would to predators (Blumstein, 2003; Beale and Monaghan, 2004a), even non-lethal human disturbances—e.g. tourism or other recreational activities—can elicit potentially costly antipredator responses (Frid and Dill, 2002; Beale and Monaghan, 2004b). For example, human presence can lead to displacement from prime breeding and foraging areas, reduced time for feeding or resting and lower reproductive success (Gander and Ingold, 1997; Beale and Monaghan, 2004b; Kerbiriou *et al.*, 2009; Bötsch *et al.*, 2017). Interestingly, however, wildlife responses to human disturbance are not uniformly negative (reviewed in Lunney *et al.*, 2008; Coetzee and Chown, 2016; Tablado and Jenni, 2017). Examples of positive effects include habituation to non-lethal human stimuli that can lower costly stress responses during critical periods like reproduction (Ferrari *et al.*, 2009; Samia *et al.*, 2015; Mohring *et al.*, 2025) and even shelter from human-averse predators (Berger, 2007; Shannon *et al.*, 2014; Hentati-Sundberg *et al.*, 2021, 2023). Despite the potential importance of this so-called ‘scarecrow effect’ (Leighton *et al.*, 2010; Kuijper *et al.*, 2015), it remains unclear whether this shield reduces perceived predation risk for prey. In that context, comparing risk perception of individuals from the same species under different magnitudes of human disturbance and natural predation risk can provide insight into the adaptation of wildlife to anthropogenic landscapes.

Predators elicit stress responses in prey, and these responses are mediated by the hypothalamo–pituitary–adrenal (HPA) axis, which stimulates the secretion of glucocorticoids [GC; e.g. corticosterone (CORT) in birds]. Baseline GC levels are often related to the energetic status of individuals (Kitaysky *et al.*, 1999; Lynn *et al.*, 2003; Angelier *et al.*, 2015; Krause *et al.*, 2017), and a moderate increase in GC levels aims to restore homeostasis (Wingfield *et al.*, 1998; Wingfield, 2003; Romero, 2004; Romero *et al.*, 2009) through metabolic and behavioural changes (Astheimer *et al.*, 1992; Landys *et al.*, 2006; Jimeno *et al.*, 2018). Such an increase in GC levels enables the restoration of energetic balance (Sapolsky *et al.*, 2000), but it can also support reproductive effort (Bonier *et al.*, 2009; Mohring *et al.*, 2023) through increased foraging activity (Angelier *et al.*, 2007; Crossin *et al.*, 2012), energy expenditure (Rivers *et al.*, 2017) and metabolic rates (Astheimer *et al.*, 1992; Jimeno *et al.*, 2018). The rapid and substantial secretion of GC following the exposure to a stressful stimulus (e.g. a predation attempt) reflects an acute stress response (Wingfield *et al.*, 1998; Angelier and Wingfield, 2013; Gormally and Romero, 2020; Grindstaff *et al.*, 2022), and elevated stress-induced GC levels activate an emergency life-history stage (ELHS) through binding to low-affinity GC receptors (Wingfield *et al.*, 1998; Angelier and Wingfield, 2013). Thus, HPA-axis sensitivity to stressors may serve as a proxy for parental investment (Wingfield and Sapolsky, 2003; Angelier and Wingfield, 2013). The link between the GC levels and the risk of predation has been investigated (Creel *et al.*, 2009; Fontaine *et al.*, 2011; Vitousek *et al.*, 2014; Mohring *et al.*, 2023), but to our knowledge, no study has tested whether a human ‘scarecrow effect’ relates to variation in GC levels in wild vertebrates.

In addition to GCs, other hormones might also influence how individuals adjust their parental effort to perceived predation risk. Thyroid hormones [THs, triiodothyronine (T3) and thyroxine (T4)] are involved in development, reproduction, thermoregulation and metabolic processes in birds (Decuypere *et al.*, 2005; McNabb, 2007; Ruuskanen *et al.*, 2021; Ruuskanen and Hsu, in press), and TH levels can be positively correlated with basal metabolic rate (BMR) under some circumstances (Chastel *et al.*, 2003; Criscuolo *et al.*, 2003; Vézina *et al.*, 2009; Elliott *et al.*, 2013; Welcker *et al.*, 2013). BMR is thought to be involved in energy-management strategies (Nilsson, 2002; Broggi *et al.*, 2019; Halsey *et al.*, 2019) that are influenced by predation risk (Biro and Stamps, 2010; Mathot and Dingemanse, 2015). Although T3 levels do not seem to be affected by acute stress (Angelier *et al.*, 2016a), the regulation of TH levels could underlie adaptive metabolic changes in response to chronic predation risk. However, to our knowledge, this has not been examined in wild vertebrates. In addition, prolactin (PRL) is a pleiotropic hormone that is central to the expression of parental care behaviour (Buntin, 1996; Smiley, 2019; Angelier, 2024). Circulating PRL is positively associated with parental effort (Angelier and Chastel, 2009; Angelier *et al.*, 2016b), but it can be reduced under environmental stress (Angelier and Chastel, 2009), with low PRL levels triggering nest desertion (Groscolas *et al.*, 2008; Spée *et al.*, 2010). Although reproductive effort is thought to vary with predation risk (Sih, 1994), the link between PRL and predation risk remains little studied (but see Mohring *et al.*, 2021, 2024). Overall, by comparing multiple and complementary hormonal markers (CORT, TH and PRL) in breeding birds facing contrasting predation risk, we can link individual variation in hormonal profiles to changes in reproductive effort in response to perceived predation risk. This is particularly relevant given that several studies have documented physiological responses to human disturbance even in the absence of observable behavioural responses (Beale and Monaghan, 2004b; Coetzee and Chown, 2016).

Common eiders *Somateria mollissima* (hereafter, eiders) breeding in the Baltic Sea provide an ideal framework to investigate endocrine responses of prey to natural predation risk and human disturbance—potentially acting as a ‘shield’ against predators. Eiders are facing the recovery of their native predator, the white-tailed eagle *Haliaeetus albicilla*, and incubating females are particularly vulnerable to predation (Öst *et al.*, 2018, 2022; Ramula *et al.*, 2018). Despite the often adverse effects of human presence at seabird colonies (Beale and Monaghan, 2004b), it may reduce eagle activity and deter eagles from breeding seabirds (Hentati-Sundberg *et al.*, 2021, 2023). In this study, we compared the endocrine profiles of female eiders breeding at two nearby colonies located in southwestern Finland. Despite their geographical proximity, the two sites drastically differ in predation risk, as one is located in a restricted-access nature reserve characterized by low human disturbance (hence high predation risk), whereas the other is characterized by intense human disturbance (tourist activities and active human predator deterrence), which largely reduces eagle presence and activity (see Materials and Methods for more details). Under the hypothesis of a human scarecrow effect, we predicted that females in the high-disturbance/low-predation colony would exhibit downregulated sensitivity of the HPA axis (i.e. lower stress-induced CORT) and lower BMR (i.e. lower T3 as a proxy) compared to those at the low-disturbance/high-predation site. We also predicted that eiders in the high-disturbance/low-predation colony would increase reproductive investment, reflected in higher baseline CORT and PRL levels, owing to reduced perceived predation risk. Finally, we examined covariation among measured hormones under contrasting predation risk to evaluate whether multiple endocrine pathways jointly mediate the response of wild birds to perceived predation risk.

## Materials and Methods

### Study system

**Figure 1:**
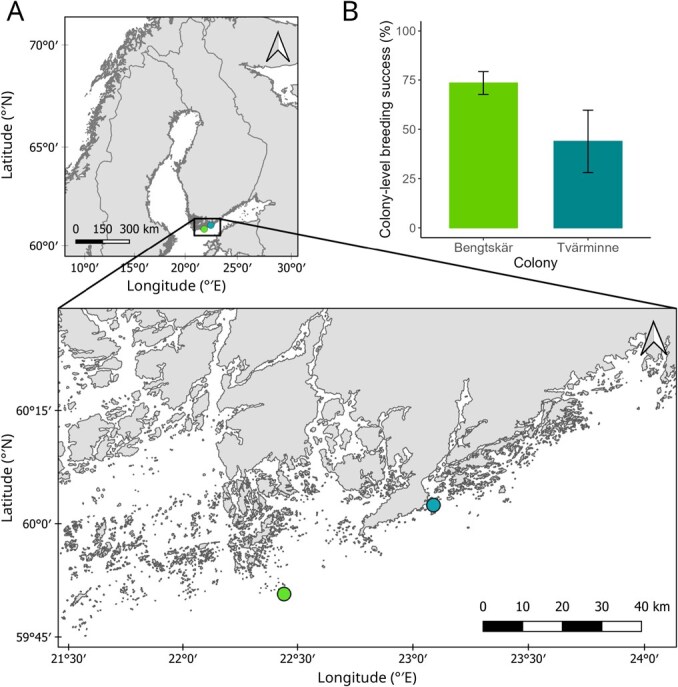
(**A**) Location of the two common eider study colonies (Bengtskär in green and Tvärminne in blue) and (**B**) average colony-specific percentage of breeding success during the years 2020, 2021 and 2023 (years with breeding success data available for both colonies). Error bars depict standard deviation.

The study was conducted at two eider colonies located ca. 40 km apart in southwestern Finland, Baltic Sea (Fig. 1A), between 16 May and 21 June 2023. The first colony was located in the archipelago surrounding Tvärminne Zoological Station (hereafter, Tvärminne; 59°50′N, 23°15′E), on islands belonging to a nature reserve, where visits are only allowed for research purposes in order to limit human disturbance. There, islands vary in size (*n* = 14 islands, mean area ± SD = 3.19 ± 3.28 ha, min = 0.13 ha, max = 10.22 ha) and are relatively close to each other, typically only hundreds of metres apart, in a total study area of ca. 15 km^2^. This colony has been shown to suffer from high predation pressure (Öst *et al.*, 2018, 2022; Jaatinen *et al.*, 2022), and survival of breeding females is lower than in most other eider populations (Ekroos *et al.*, 2012b; Tjørnløv *et al.*, 2020). The second colony was on Bengtskär (59°44′N, 22°30′E), a rocky island of about 1.5 ha isolated from the mainland (ca. 25 km from the mainland). This island has turned into a popular tourist destination since the opening of its lighthouse as a museum in 1995. As a consequence of the presence of a human settlement on the island, active deterrence methods against avian predators by the lighthouse staff around the clock and daily influxes of tourists, human-induced disturbance shields the eiders from eagles. This has led to a sharp increase in the number of eiders breeding at Bengtskär (Vösa *et al.*, 2017), contrasting with the overall population decline witnessed in the Baltic Sea (Ekroos *et al.*, 2012a; Desholm *et al.*, 2013; Lehikoinen *et al.*, 2022). Due to the small area of Bengtskär and the increase in the size of its eider colony, nest density is substantially higher at Bengtskär (230 nests per hectare in 2023) than at Tvärminne (36 nests per hectare on the island with the highest nest density in 2023). Hatching success is also higher at Bengtskär than at Tvärminne [Fig. 1B; Bengtskär: 25 out of 35 nests, 71.4%; Tvärminne: 113 out of 214, 52.8%; binomial generalized linear model (LM): χ^2^₁ = 4.38, *P* = 0.036]. At Bengtskär, these numbers are based on a representative subsample of nests monitored in 2023, whereas at Tvärminne, they include all monitored nests in 2023.

Incubating female eiders were caught on their nest with hand nets (*n*_Tvärminne_ = 82, *n*_Bengtskär_ = 22, *n*_total_ = 104). Females incubate ca. 26 days (Korschgen, 1977), and the trapping period was timed to correspond to the later phase of incubation in order to minimize the risk of nest abandonment (Bolduc and Guillemette, 2003). All trapped females were ringed with a unique metal ring upon capture and weighed to the nearest 5 g with a Pesola spring balance, and radius-ulna length was measured to the nearest 1 mm with a wing ruler as a proxy of structural size. We observed a difference in body mass between the birds sampled in the two colonies (two-sample *t* test, *t* = 2.71, *df* = 36.18, *P* = 0.01), with lighter birds at Bengtskär, compared to Tvärminne (average body mass difference = 105 g). However, eiders fast throughout incubation and may lose up to 46% of their body mass during this period (Parker, 1990), with an average daily mass loss of 25 g/day (Garbus *et al.*, 2018). Because the exact incubation stage at capture was not precisely known for all females, we cannot distinguish whether the lower body mass of Bengtskär birds reflects poorer body condition or simply later sampling during incubation; the observed 105-g difference in body mass corresponds to approximately 4.2 days of fasting at 25 g/day (Garbus *et al.*, 2018). Given this uncertainty, we controlled for body mass in our analyses. Body mass was preferred over a scaled mass index adjusted for structural size because, in female eiders, body mass primarily reflects fasting-related mass loss, and there was no correlation between body size and body mass in our dataset (Pearson’s correlation: *r* = 0.003, *P* = 0.98). In addition, clutch size was recorded. There was no difference in clutch size between the two colonies (two-sample *t* test, *t* = 0.21, *df* = 20.85, *P* = 0.83); hence, clutch size was not included as a covariate in the analyses. Handling of animals was reviewed and approved by the National Animal Experiment Board in Finland (permit numbers ESAVI/1697/04.10.03/2012, ESAVI/2831/04.10.07/2015, ESAVI/4053/2018, ESAVI/10022/2021, ESAVI/9500/2021) and the Finnish Wildlife Agency EH41D-00001-2022 and UU41D_0001_2023, and complied with the regulations of Tvärminne Zoological Station and the permissions issued from Bengtskär Oy.

### Hormone assays

In total, 104 female eiders (*n*_Tvärminne_ = 82, *n*_Bengtskär_ = 22) were sampled for blood twice from the ulnar vein. At least 1.5 ml of blood was sampled from all females, enabling the measurement of baseline and stress-induced CORT levels, as well as baseline PRL levels. T3 and T4 levels were measured in a subset of these females (*n*_Tvärminne_ = 9, *n*_Bengtskär_ = 22, *n*_total_ = 31), as part of a separate study. For all individuals, the first blood sample was taken within 3 min of capture (mean ± SD = 2 min 52 s ± 10 s; range, 1 min 49 s to 2 min 59 s) in order to measure baseline levels of CORT (Romero and Reed, 2005), PRL, T3 and T4. Each female was kept in hands, and the second blood sample was taken 18 ± 3 min on average following capture (range, 11–28 min) in order to measure stress-induced CORT levels. We verified that stress-induced CORT levels were not linked to sampling time in both colonies (linear regression, Tvärminne: *t* = −0.45, *P* = 0.66; Bengtskär: *t* = 0.40, *P* = 0.70). Blood samples were centrifuged and plasma samples were stored at −20°C until assay. Plasma concentrations of CORT and PRL were measured at the Centre d’Etudes Biologiques de Chizé (France). CORT was assayed by ELISA using a commercial kit supplied by Demeditec. The assay has been validated for eiders by performing linearity and spiking tests. PRL was assayed by radioimmunoassay, as previously described and validated for the Baltic Sea eider population (Criscuolo *et al.*, 2002; Mohring *et al.*, 2021, 2024). Samples were run in duplicate (20 μl for CORT and 25 μl for PRL). Eight assays were run for CORT (inter-assay CV: 10.95%, intra-assay CV: 7.38%) and one for PRL (intra-assay CV: 5.84%). Limits of detection of CORT and PRL assays were 0.20 and 0.62 ng/ml, respectively. T3 and T4 were analysed at the University of Turku and Åbo Akademi University (Finland) following a described extraction protocol (Ruuskanen *et al.*, 2016) and nano-LC/MS/MS protocol of measurement (Ruuskanen *et al.*, 2018; Hsu *et al.*, 2022). We used ^13^C_12_-T4 as reference standard for recovery correction, and ^13^C_6_-T3 and ^13^C_6_-T4 were used as internal standards for T3 and T4 (limit of detection: 5.9 amol for T3 and 3.5 amol for T4, limit of quantification: 10.6 and 17.9 amol, respectively; Ruuskanen *et al.*, 2018).

### Statistical analyses

All statistical analyses were conducted in R 4.3.1. (R Core Team, 2023). All continuous variables were centred and scaled. We checked that all models met the assumptions of normality and homoscedasticity of residuals.

First, we compared the physiological profiles of female eiders sampled at the two colonies. To account for the non-independence of baseline and stress-induced corticosterone levels, we built a linear mixed model (LMM) with corticosterone levels as the dependent variable and sample type (baseline or stress-induced), colony and a two-way interaction between sample type and colony as independent variables. Body mass was also included as a covariate to control for the energetic state of female eiders. Female identity was included as a random intercept to account for the non-independence of baseline and stress-induced corticosterone levels measured in the same female. The model was implemented with the *lmer* function (R package ‘lme4’; Bates *et al.*, 2015). We built three additional LMs with baseline PRL levels, T3 concentrations and T4 concentrations as dependent variables, respectively, and colony as the independent variable. Body mass was also included as a covariate in all LMs to control for the energetic state of female eiders. To check the robustness of our results, we re-ran the aforementioned LMs and LMMs with a three-way interaction between colony, sample type and body mass (LMM) or a two-way interaction between colony and body mass (LMs; see Supplementary Materials  S1 and S2).

Second, we explored the potential covariation among the focal hormones and tested whether it differed between colonies. Under elevated predation risk, reproductive investment is often reduced (Eggers *et al.*, 2006; Travers *et al.*, 2010; Zanette *et al.*, 2011), potentially via endocrine mechanisms (Angelier and Chastel, 2009). Importantly, increased predation risk may filter which individuals persist and breed (e.g. selective disappearance of lower-quality individuals), a process that may operate also in eiders (Mohring *et al.*, 2022; Öst *et al.*, 2022). Such filtering could narrow phenotypic and physiological variation and thereby alter the strength of covariation among hormones. Relationships between the different hormones were investigated in separate sets of models to maximize sample size, due to the relatively small sample size for THs. To do so, we first looked at the colony-specific relationship between baseline PRL and CORT levels, by fitting LMs with baseline PRL levels as the dependent variable and baseline CORT levels, colony and the two-way interaction between baseline CORT levels and colony as independent variables. Similarly, we looked at the relationships between stress-induced CORT and baseline PRL levels, and between the magnitude of increase in CORT in response to an acute stressor ∆CORT (difference between stress-induced CORT and baseline CORT levels) and baseline PRL levels. Accordingly, we built LMs with baseline PRL levels as the dependent variable and stress-induced CORT levels (respectively, ∆CORT), colony and the two-way interaction between stress-induced CORT levels (respectively, ∆CORT) and colony as independent variables. Additionally, we assessed the relationships between THs and CORT and PRL. We built two sets of LMs with T3 or T4 as dependent variables and baseline CORT levels (stress-induced CORT, ∆CORT or baseline PRL levels, respectively), colony and its two-way interaction with baseline CORT levels (stress-induced CORT, ∆CORT or baseline PRL levels, respectively) as independent variables. To explore the association between THs in each colony, we built a model with T3 as the dependent variable and T4, colony and its two-way interaction with T4 as independent variables. For each LM, we used a backward stepwise approach (Anderson and Burnham, 2004) to select the most parsimonious model by progressively eliminating non-significant terms (*P* > 0.100). Because these analyses were based on small, biologically pre-specified candidate models, rather than on a large exploratory model set, we considered hierarchical backward simplification to be a justified approach, particularly given the limited sample size for the TH subset. When the two-way interaction term was retained in the most parsimonious model, post hoc comparisons and estimates of slopes were performed with the *emtrends* function (R package ‘emmeans’; Lenth, 2024) to assess the statistical significance of slopes and the parameter estimate of the trend in each colony.

## Results

### Colony-specific variation in hormonal profiles

**Figure 2:**
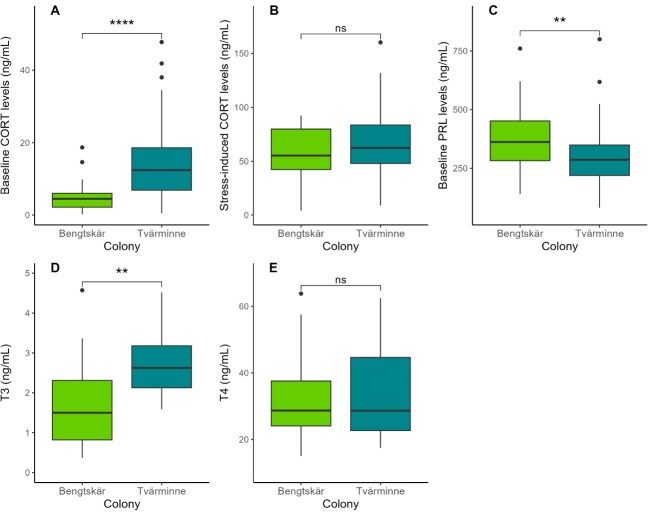
Differences in (**A**) baseline corticosterone (CORT) levels, (**B**) stress-induced corticosterone levels, (**C**) baseline prolactin (PRL) levels, (**D**) triiodothyronine (T3) levels and (E) thyroxine (T4) levels between female common eiders breeding at Bengtskär (high human disturbance and low predation risk) and at Tvärminne (low human disturbance and high predation risk).

CORT levels significantly varied between colonies, with higher levels at Tvärminne than at Bengtskär (Table 1, Fig. 2A), and stress-induced CORT levels were higher than baseline CORT levels (Table 1). However, the magnitude of the stress response was similar in the two colonies (sample type × colony interaction term; Table 1, Fig. 2B). In contrast, baseline PRL levels were significantly higher in eiders breeding at Bengtskär than at Tvärminne (Table 2, Fig. 2C). The colonies significantly differed in T3 levels, but not in T4 levels (Table 2, Fig. 2D and E), with eiders breeding at Tvärminne displaying higher T3 levels (Table 2, Fig. 2D). We did not detect any significant association between hormone levels and body mass (Tables 1 and 2). In addition, supplementary analyses, including the interactions between colony and body mass, did not materially alter the results, indicating that the associations between body mass and hormone levels did not differ between colonies (Supplementary Materials  S1 and S2).

**Table 1:** LMM testing for colony differences in corticosterone levels and in the magnitude of the corticosterone stress response among incubating female common eiders from two colonies (Bengtskär and Tvärminne), controlling for body mass

Dependent variable	Independent variable	χ^2^	*P*	*n*	*n* _T_	*n* _B_
CORT	**Colony**	**4.814**	**0.028**	100	79	21
	**Sample type**	**389.619**	**<0.001**			
	Colony × sample type	0.072	0.788			
	Body mass	0.642	0.423			

*n*, total sample size; *n*_T_, sample size at Tvärminne; *n*_B_, sample size at Bengtskär; CORT, corticosterone levels; sample type, baseline or stress-induced corticosterone level.

Female identity was included as a random effect. Significant effects (*P* ≤ 0.05) are presented in bold.

**Table 2:** LMs testing for colony differences in each hormone (baseline prolactin and THs triiodothyronine and thyroxine) among incubating female common eiders from two colonies (Bengtskär and Tvärminne), controlling for body mass

Dependent variable	Independent variable	*F*	*P*	*n*	*n* _T_	*n* _B_
PRL	**Colony**	**5.839**	**0.018**	93	72	21
	Body mass	1.174	0.282			
T4	Colony	0.995	0.327	31	9	22
	Body mass	0.410	0.527			
T3	**Colony**	**8.922**	**0.006**	31	9	22
	Body mass	1.121	0.299			

*n*, total sample size; *n*_T_, sample size at Tvärminne; *n*_B_, sample size at Bengtskär; PRL, baseline prolactin levels; T3, triiodothyronine levels; T4, thyroxine levels.

Significant effects (*P* ≤ 0.05) are presented in bold.

**Table 3:** Full LMs and the associated most parsimonious LMs selected using a backward stepwise selection procedure

Full model	n	n_T_	n_B_	Most parsimonious model
PRL ~ CORT_b_ + Colony + CORT_b_ × Colony	93	72	21	PRL ~ Colony
PRL ~ CORT_stress_ + Colony + CORT_stress_ × Colony	90	69	21	PRL ~ Colony
PRL ~ ∆CORT + Colony + ∆CORT × Colony	90	69	21	PRL ~ Colony
T3 ~ CORT_b_ + Colony + CORT_b_ × Colony	30	9	21	T3 ~ CORT_b_ + Colony + CORT_b_ x Colony
T3 ~ CORT_stress_ + Colony + CORT_stress_ × Colony	29	8	21	T3 ~ CORT_stress_ + Colony + CORT_stress_ x Colony
T3 ~ ∆CORT + Colony + ∆CORT × Colony	29	8	21	T3 ~ Colony
T3 ~ PRL + Colony + PRL × Colony	29	8	21	T3 ~ Colony
T4 ~ CORT_b_ + Colony + CORT_b_ × Colony	30	9	21	T4 ~ 1
T4 ~ CORT_stress_ + Colony + CORT_stress_ × Colony	29	8	21	T4 ~ CORT_stress_
T4 ~ ∆CORT + Colony + ∆CORT × Colony	29	8	21	T4 ~ **∆**CORT
T4 ~ PRL + Colony + PRL × Colony	29	8	21	T4 ~ 1
T3 ~ T4 + Colony + T4 × Colony	31	9	22	T3 ~ T4 + Colony

*n*, total sample size; *n*_T_, sample size at Tvärminne; *n*_B_, sample size at Bengtskär; PRL, baseline prolactin levels; CORT_b_, log-transformed baseline corticosterone levels; CORT_stress_, stress-induced corticosterone levels; ∆CORT, difference between stress-induced and baseline corticosterone levels; T3, triiodothyronine levels; T4, thyroxine levels.

LMs explore the potential covariation among the focal hormones (baseline prolactin, baseline and stress-induced corticosterone, ∆CORT and THs triiodothyronine and thyroxine) and tested whether it differed among incubating female common eiders from the two colonies (Bengtskär and Tvärminne).

### Relationships between hormones in the two colonies

**Figure 3:**
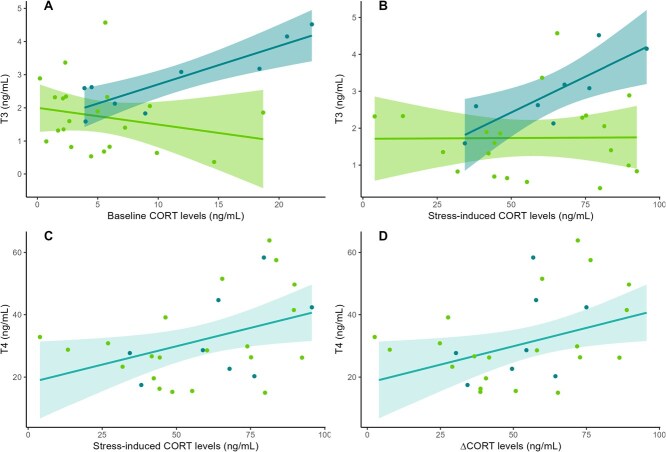
Relationship between (**A**) triiodothyronine (T3) and baseline corticosterone (CORT) levels, (**B**) T3 and stress-induced CORT levels, (**C**) thyroxine (T4) and stress-induced CORT levels and (**D**) thyroxine (T4) and ∆CORT levels in female common eiders breeding at Bengtskär (high human disturbance and low predation risk, in green) and at Tvärminne (low human disturbance and high predation risk, in blue). Regression lines are presented in light blue when the relationship does not differ between the two colonies and in green (Bengtskär) or medium blue (Tvärminne) for colony-specific relationships. Shaded areas account for 95% confidence intervals.

We did not detect any significant relationship between baseline PRL levels and baseline or stress-induced CORT levels or ∆CORT (Table 3). Regarding THs, the association between baseline CORT and T3 differed between colonies (significant CORT × colony interaction; Table 3, Fig. 3A). This interaction reflected a significant positive association between T3 and baseline CORT levels at Tvärminne (*E* ± SE = 0.116 ± 0.044, *t* = 2.639, *P* = 0.014; Table 4, Fig. 3A), but no such association at Bengtskär (*E* ± SE = −0.051 ± 0.045, *t* = −1.130, *P* = 0.269; Table 4, Fig. 3A). The relationship between T3 levels and stress-induced CORT levels was positive at Tvärminne (*E* ± SE = 0.039 ± 0.018, *t* = 2.164, *P* = 0.040, Table 4, Fig. 3B) but not at Bengtskär (*E* ± SE = 0.001 ± 0.009, *t* = 0.048, *P* = 0.962, Table 4, Fig. 3B), though the two-way interaction between stress-induced CORT levels and colony on T3 levels was only marginally significant (Table 4). However, a similar relationship was not supported between T3 levels and ∆CORT (Table 3). Furthermore, we did not detect any significant relationship between T3 and baseline PRL levels or the two-way interaction between baseline PRL levels and colony (Table 3). In addition, we did not detect any significant relationship between T4 levels and baseline CORT or PRL levels (Table 3). However, we observed a significant positive relationship between stress-induced CORT levels and T4 levels, as well as between ∆CORT and T4 levels (stress-induced CORT: *E* ± SE = 0.235 ± 0.101, *t* = 2.339, *P* = 0.027; ∆CORT: *E* ± SE = 0.230 ± 0.108, *t* = 2.126, *P* = 0.043, Table 4, Fig. 3C and D), and this association did not differ between colonies (Table 3). Finally, T3 levels were positively associated with T4 levels (*E* ± SE = 0.029 ± 0.012, *t* = 2.377, *P* = 0.025, Table 4), and this relationship did not differ significantly between colonies (Table 3).

**Table 4:** Most parsimonious LMs selected for each hormone (baseline prolactin, baseline and stress-induced corticosterone, ∆CORT and THs triiodothyronine and thyroxine) using a backward stepwise selection approach, testing for colony-specific covariation among the focal hormones in female common eiders breeding at Bengtskär and Tvärminne

Dependent variable	Independent variable	*F*	*P*
PRL	**Colony**	**7.469**	**0.008**
T3	**CORT**_**b**_	**1.197**	**0.284**
	**Colony**	**4.842**	**0.037**
	**CORT**_**b**_ × **Colony**	**7.046**	**0.013**
	CORT_stress_	0.950	0.339
	**Colony**	**8.363**	**0.008**
	***CORT***_***stress***_ × ***Colony***	***3.737***	***0.065***
	**T4**	**5.234**	**0.025**
	**Colony**	**6.833**	**0.011**
T4	**CORT**_**stress**_	**5.472**	**0.027**
	**∆CORT**	**4.521**	**0.043**

PRL, baseline prolactin levels; CORT_b_, log-transformed baseline corticosterone levels; CORT_stress_, stress-induced corticosterone levels; ∆CORT, difference between stress-induced and baseline corticosterone levels; T3, triiodothyronine levels; T4, thyroxine levels.

Significant effects (*P* ≤ 0.05) are presented in bold and nearly significant ones (*P* ≤ 0.10) in bold italics.

## Discussion

In this study, we compared endocrine profiles of incubating female eiders breeding in two geographically proximate colonies markedly differing in levels of human disturbance and predation pressure. By comparing multiple and complementary hormonal markers, as proxies of energy expenditure and parental care (CORT, TH and PRL), we tested whether human disturbance—by deterring natural predators—reduces the level of threat perceived by prey (the human ‘scarecrow effect’; Leighton *et al.*, 2010; Kuijper *et al.*, 2015) and whether the reduced threat favours increased reproductive investment, as predicted by life-history theory (Stearns, 1992; Ghalambor and Martin, 2001). Overall, support for the human ‘scarecrow effect’ and the predicted shift in reproductive investment was partial and hormone dependent. Consistent with our prediction of reduced energetic expenditure in the high-disturbance/low-predation colony, we observed lower T3 levels in incubating females from that colony. Although we predicted higher baseline CORT levels—supporting higher reproductive effort—in the human-protected colony, levels were higher in the colony facing elevated predation pressure. This result suggests that baseline CORT levels may rather reflect a higher energetic cost of incubating under elevated predation risk. Contrary to our a priori prediction, stress-induced CORT levels did not differ between colonies, suggesting that eiders maintain sensitivity to acute stressors despite a human ‘scarecrow effect’. Finally, consistent with our prediction, incubating females in the high-disturbance/low-predation colony showed higher baseline PRL levels, suggesting greater parental effort. Our results show that the three hormonal axes did not covary in a single direction. This underscores the value of using complementary endocrine markers and cautions against interpreting baseline CORT as a straightforward proxy for reproductive investment under energetic constraints without accounting for prevailing predation risk.

### Variation in TH levels in contrasting breeding environments

We found that eiders displayed higher T3 levels at Tvärminne than at Bengtskär. T3 is considered the active form of THs (Darras *et al.*, 2006; Hsu *et al.*, 2022), due to its well-known functions as a transcription factor (Darras, 2022). T3 has been shown to be positively linked to BMRs in several seabirds (Elliott *et al.*, 2013; Welcker *et al.*, 2013), as well as passerines (Chastel *et al.*, 2003). In fasting birds such as eiders, there is experimental evidence for a positive association between T3 levels and energy expenditure during incubation (Criscuolo *et al.*, 2003; Bourgeon and Raclot, 2007). The higher T3 levels observed at Tvärminne can reflect higher BMRs and higher energy expenditure during incubation. Interestingly, while T3 levels were higher at Tvärminne, we did not detect any significant difference in T4 levels across the two colonies. This finding suggests that eiders breeding at Tvärminne may begin incubation with higher T4 levels and may convert T4 into T3 more rapidly than females breeding at Bengtskär. Higher conversion of T4 into T3 under higher predation threat (i.e. at Tvärminne) may thus represent high maintenance costs and energy expenditure required to cope with harsh environments (Swanson *et al.*, 2017). In addition, previous studies on birds have reported that high BMRs were associated with longer escape distance from an approaching human (Møller, 2009)—commonly perceived by prey as a predation attempt (Frid and Dill, 2002)—and with energy-costly behaviours across taxa (Mathot *et al.*, 2019). In addition, experimentally supplementing convict surgeonfish *Acanthurus triostegus* with T3 increased their ability to avoid predation (Besson *et al.*, 2020). Maintaining high BMRs—mediated by high T3 levels—may prove adaptive when breeding under high predation pressure (Møller, 2009). In further support of this hypothesis, we observed a positive correlation between T3 and stress-induced CORT levels, reflecting higher acute stress. This relationship was only evident at Tvärminne, where predation threat is higher. In light of evidence that risk-averse individuals show a systematically potentiated HPA axis (Baugh *et al.*, 2017), this result suggests that individuals may differ in phenotypes, with reactive phenotypes being characterized by elevated levels of stress-induced CORT and T3. Nevertheless, because this pattern was not supported when ΔCORT was used instead of stress-induced CORT, the relationship between T3 and acute HPA responsiveness should be interpreted cautiously.

### Baseline CORT levels, perceived level of threat and reproductive costs

Although we predicted higher baseline CORT levels in the high-disturbance/low-predation colony, baseline CORT levels were lower at Bengtskär than at Tvärminne. Given that baseline CORT in Tvärminne is positively related to breeding success, clutch size and mass (Mohring *et al.*, 2021, 2023), our result may indicate higher energetic investment in reproduction at Tvärminne. While clutch size did not differ between colonies, higher predation at Tvärminne could promote selective disappearance of poor-quality individuals with a low reproductive investment, thereby elevating mean baseline CORT levels relative to Bengtskär. In support of this hypothesis, the population of eiders breeding at Tvärminne has been declining over the past decades (Öst *et al.*, 2022), and previous studies have highlighted a decrease in female eider breeding propensity (Öst *et al.*, 2018), an ageing of the population (Mohring *et al.*, 2022; Öst *et al.*, 2022) and an increase in body condition of breeders (Öst *et al.*, 2018, 2022). Older, more experienced females may invest more in reproduction than younger ones (Coulson, 1999), and larger body reserves may, similarly, allow individuals to allocate more resources to reproduction (Hennin *et al.*, 2018). However, we did not find a significant correlation between baseline CORT and PRL levels—mediating parental care behaviour and parental effort (Buntin, 1996; Angelier and Chastel, 2009).

Given that a moderate elevation of baseline CORT levels aims to restore energetic balance within an organism (Wingfield *et al.*, 1998; Wingfield, 2003; Romero, 2004; Romero *et al.*, 2009), it can both arise to help the organism support higher reproductive effort (Bonier *et al.*, 2009; Mohring *et al.*, 2023) or cope with chronic environmental stressors, including predation risk (Scheuerlein *et al.*, 2001; Clinchy *et al.*, 2004; Travers *et al.*, 2010; Angelier and Wingfield, 2013). It is therefore important to consider alternative interpretations of colony-level differences. One alternative interpretation is the higher energetic cost of incubating under predation threat, which can elevate baseline CORT under chronic stress (Scheuerlein *et al.*, 2001; Clinchy *et al.*, 2004; Travers *et al.*, 2010; Angelier and Wingfield, 2013). Supporting this idea, baseline CORT levels covaried positively with both stress-induced CORT levels (Pearson’s correlation: *r* = 0.33, *P* < 0.001) and—at Tvärminne—with T3 levels, a marker of higher energy expenditure during incubation (Criscuolo *et al.*, 2003; Bourgeon and Raclot, 2007). In addition, periodic disturbance by white-tailed eagles may alter incubation behaviour by increasing recess frequency, which could, in turn, modify baseline CORT levels. As prolonged incubation bouts are associated with dehydration (Bourne *et al.*, 2021), predator-induced recesses may provide females with opportunities to drink and rehydrate. Because dehydration may elevate baseline CORT levels (Brischoux *et al.*, 2020; Brusch *et al.*, 2020), more frequent recesses could reduce dehydration-induced elevation of baseline CORT. However, osmoregulatory processes associated with saltwater intake in marine environments have also been linked to elevated baseline CORT levels (Brischoux *et al.*, 2015). The net effect of predator-induced recesses on baseline CORT is therefore difficult to predict and warrants further study combining behavioural observations, hydration state, osmoregulatory measures and individual CORT profiles.

In addition to human disturbance and predation regime, the two colonies also differ in habitat structure. Bengtskär is an exposed, treeless rocky island, whereas the Tvärminne colony mainly consists of forested islands. Such habitat differences could influence endocrine profiles not only through differences in concealment from predators, but also through exposure to wind and weather, which may alter nest microclimate and potentially affect incubation energetics (Kilpi and Lindström, 1997), although the proposed link to incubation energetics has since been questioned (Öst *et al.*, 2008). Previous work from Tvärminne showed that the relationship between baseline CORT and adult predation risk was habitat specific: on open islands, baseline CORT declined with increasing predation risk, whereas females on forested islands appeared less sensitive to such variation (Mohring *et al.*, 2023). This suggests that vegetation cover can modify how predation risk is translated into endocrine state. Importantly, however, these earlier findings point to an interaction between habitat structure and predation risk, rather than a simple main effect of vegetative cover. Habitat openness alone is therefore unlikely to explain the present colony-level differences in baseline CORT profiles.

### Similarity in acute stress responses under high human disturbance or predation risk

Despite colony-level differences in baseline CORT levels, we did not detect any significant difference in stress-induced CORT levels—or in the magnitude of increase in CORT levels in response to a stressor—between eiders breeding at Bengtskär and Tvärminne. This result is surprising given the large difference in predation by white-tailed eagles between the two sites. It could indicate a similar perception of risk at both colonies, either due to high human disturbance (Bengtskär) or high predation risk (Tvärminne), although this interpretation seems unlikely. Given the pronounced differences in other hormonal markers between colonies, alternative hypotheses also have to be considered.

First, because the activation of the ELHS prioritizes survival over reproduction, dampening stress-induced CORT levels may be maladaptive when exposed to imminent threat (Wingfield and Sapolsky, 2003; Landys *et al.*, 2006; Angelier and Wingfield, 2013). Therefore, even if eiders at Bengtskär perceive a reduced level of risk due to a human scarecrow effect, dampening GC stress responses may impair an individual’s ability to effectively respond to an acute threat (Wingfield *et al.*, 1998; Wingfield and Sapolsky, 2003; Angelier and Wingfield, 2013), potentially increasing mortality risk when confronted with a real predator. Moreover, any predator ‘refuge’ created by human presence at Bengtskär is spatially and temporally restricted to the breeding colony. Females still experience predation risk outside this context across much of the annual cycle, making downregulation of the acute stress response during the short breeding season less likely, and potentially costly for year-round survival. This interpretation is consistent with the ‘classic’ preparative hypothesis (Sapolsky *et al.*, 2000): acute elevations of GCs can prime individuals for upcoming challenges, not just the immediate stressor. Our results are consistent with findings in chick-rearing Adélie penguins *Pygoscelis adeliae*, where individuals exposed to varying levels of human disturbance nonetheless exhibited similar stress-induced CORT levels (Marciau *et al.*, 2023). Alternatively, but non-exclusively, the increase in CORT levels in response to a stressor has inhibitory effects on PRL secretion (Angelier *et al.*, 2009), and the activation of ELHS can lead to the reduction or suppression of reproductive activities (Wingfield and Sapolsky, 2003; Angelier and Wingfield, 2013; Vitousek *et al.*, 2014). Individual plastic dampening of HPA axis sensitivity or selective disappearance of individuals displaying heightened stress responses at Tvärminne may therefore also explain the lack of detectable colony-level differences in stress-induced CORT levels.

Stress-induced CORT levels may also show lower within-individual plasticity than baseline CORT levels. Supporting this idea, stress-induced CORT levels have been shown to be repeatable (Schoenemann and Bonier, 2018), heritable (Béziers *et al.*, 2019) and linked to consistent individual stress-coping styles (Baugh *et al.*, 2017). In line with this, prior work suggests that female eiders may choose nest sites matching their physiological (D’Alba *et al.*, 2011) or behavioural (Mohring *et al.*, 2022) stress-coping phenotype (the personality-matching hypothesis; Holtmann *et al.*, 2017), instead of modifying their stress responses in relation to local environmental conditions. The similarity in stress-induced CORT levels between colonies may arise because females choose nest sites that match their inherent stress-coping phenotypes—assumed to be similar between colonies—leading to equivalent overall stress-induced CORT profiles despite differing environments, rather than reflecting differences in local disturbance or predation risk.

Alternatively, individuals may vary in the timing of peak CORT levels in response to a stressor (Cockrem and Silverin, 2002; Broadus *et al.*, 2025). Indeed, previous studies showed that while the rise in CORT levels typically starts several minutes after capture and reaches a plateau after 15–30 min, it may still increase after 60 min for some individuals (Broadus *et al.*, 2025). Hence, even when blood samples for the acute stress response are collected within a similar time window, individuals may not be at the same stage of that response. Measuring the full stress-response curve, with CORT sampled at several time points, may therefore provide a more informative picture of how individuals respond to acute stressors. Such inter-individual variation in stress response is still poorly understood and could be linked to intrinsic factors such as previous experience with stressors or body condition (Cockrem and Silverin, 2002).

The human ‘scarecrow effect’ may decouple physiological and behavioural traits, leading to novel phenotypes (Sadoul *et al.*, 2021). Thus, even if individuals show a physiological response to disturbance, they may not exhibit the behavioural profile typically associated with high HPA-axis sensitivity (Koolhaas *et al.*, 1999), particularly under relaxed predation pressure and habituation to humans (Beale and Monaghan, 2004b; Coetzee and Chown, 2016). For example, in European starlings (*Sturnus vulgaris*), urban nestlings exposed to greater human disturbance show higher stress-induced corticosterone than rural ones, yet behavioural styles remain similar (Guindre-Parker *et al.*, 2022). In a similar manner, female eiders breeding at Bengtskär tolerate closer human approaches—i.e. display shorter flight initiation distance—than those at Tvärminne (our unpublished work), despite the observed similar HPA axis sensitivity. This response may be mediated by several mechanisms, including but not limited to behavioural habituation to human presence, visual stimulation of cooperative breeding in a dense colony (Kristjansson and Jónsson, 2015) or a compensatory role of elevated prolactin levels at Bengtskär (Angelier *et al.*, 2009; Riechert *et al.*, 2014).

### Perceived level of threat and PRL

Baseline PRL was higher at Bengtskär than at Tvärminne, consistent with higher parental effort (Angelier and Chastel, 2009; Angelier *et al.*, 2016b). This matches life-history predictions linking predation risk to reduced reproductive investment (Zanette *et al.*, 2011). In support of this hypothesis, breeding success is higher at Bengtskär than at Tvärminne. Importantly, we cannot tell apart whether this finding reflects an adaptive response to relaxed predation risk or is driven by the social and environmental context of human disturbance and higher breeding density.

In long-lived species such as eiders, parents are predicted to adjust parental investment to the perceived level of threat (Williams, 1966). In a previous study carried out on eiders breeding at Tvärminne, female baseline PRL levels were not directly related to predation pressure (Mohring *et al.*, 2024). However, maintaining high baseline PRL may promote hatching success under challenging conditions, such as poor winter conditions or high predation risk (Mohring *et al.*, 2024). Likewise, under chronic, non-lethal human disturbance, sustained parental effort (associated with elevated PRL) may reduce nest failure and abandonment, consistent with predictions that prey downregulate costly antipredator responses when repeatedly exposed to harmless stimuli (the risk-allocation hypothesis; Lima and Bednekoff, 1999; Ferrari *et al.*, 2009, 2010). In line with this, elevated baseline PRL has been associated with greater nest attendance and faster return to the nest after disturbance (Criscuolo *et al.*, 2005; Angelier *et al.*, 2009, 2015). In contrast, low PRL levels are associated with egg neglect (Angelier *et al.*, 2015) and nest desertion (Groscolas *et al.*, 2008; Spée *et al.*, 2010).

Because PRL is closely associated with commitment to incubation and parental care, variation in breeding density could influence baseline PRL both via competition and via increased exposure to conspecific breeding cues. First, maintaining high incubation constancy (mediated by elevated baseline PRL levels) may be adaptive when breeding in dense colonies with high competition for nest sites and prevalent conspecific brood parasitism (Andersson *et al.*, 2015), such as Bengtskär (Ask *et al.*, 2025). Conspecifics may take advantage of an absence from the nest to take over or parasitize the nest (Swennen *et al.*, 1993; Robertson, 1998; Andersson *et al.*, 2015). Second, frequent exposure to conspecific parental interactions may elevate PRL levels in nearby focal breeders (Beaulieu *et al.*, 2017), suggesting that conspecific parental activity can act as a social cue reinforcing parental motivation. At Bengtskär, close proximity to breeding conspecifics likely increases exposure to parental activity and visual cues of hatching broods, which could socially reinforce parental state and contribute to elevated PRL levels. Such a mechanism is not mutually exclusive with selection for high incubation constancy arising from nest-site competition and conspecific brood parasitism.

## Conclusions

We predicted that human disturbance would reduce prey perception of risk from natural predators, thereby promoting increased reproductive investment, manifested as differences in hormone profiles of female eiders from two colonies with contrasting disturbance-predation regimes. Indeed, we observed clear colony-specific hormone level differences partly aligning with our a priori predictions. Thus, females at Tvärminne (low-disturbance/high-predation colony) showed higher baseline CORT and T3 levels, whereas those at Bengtskär (high-disturbance/low-predation colony) showed higher baseline PRL levels, with no colony differences in stress-induced CORT or T4.

Despite growing interest in the association between THs and predation risk in other taxa, empirical evidence in birds remains limited and mixed. Thus, an interspecific comparative study suggested a positive correlation between metabolism and predator avoidance (Møller, 2009), though T3 was not directly measured in that study. In contrast, experimental injections of T3 and T4 to eggs led to no changes in offspring antipredator behaviours in quail (Aho *et al.*, 2023). The relationship between THs and predation threat is not straightforward and likely context dependent. Here, even with a modest sample size, we were able to show higher T3 levels in a colony facing high predation risk. In addition, we highlighted a positive association between T3 and both baseline and stress-induced CORT levels—but not ∆CORT—confined to the high-predation colony of Tvärminne. These results emphasize the need for more studies to fully uncover the elusive relationship between THs and predation risk in birds and other vertebrates alike.

Importantly, given the correlative nature of this study, we cannot determine to what extent colony differences in endocrine profiles reflect plastic physiological adjustment to human disturbance and predation risk versus selective processes. Individuals may be able to adjust their physiological responses to the degree of risk perceived in the environment or attenuate these responses through habituation (Killen *et al.*, 2013), for example, in response to benign human disturbance. Conversely, selection could favour less-sensitive phenotypes. Future work combining longitudinal sampling of individual endocrine profiles and experimental manipulations of disturbance and predation risk will be needed to disentangle plasticity from selection.

## Supplementary Material

Web_Material_coag036

## Data Availability

The datasets used in this are available from the corresponding authors on reasonable request.
