# Supplementary material for: Same species, different risks: predation shapes hormonal profiles in a breeding sea duck, the common eider
Source: Conserv Physiol. 2026 Jun 24;14(1):coag036. doi: 10.1093/conphys/coag036 (PMC13293623; doi:10.1093/conphys/coag036)
Supplement: Web_Material_coag036 [file web_material_coag036.zip › Supplementary_Materials_coag036.pdf]

Supplementary materials for:

Same species, different risks: predation shapes hormonal profiles in a breeding sea duck, the common eider

Authors: Bertille Mohring[1,2,3,*], Farshad S. Vakili[4,*], Frédéric Angelier[3], Kim Jaatinen[5], Suvi Ruuskanen[6], Olivier Chastel[3], Charline Parenteau[3], Bin-Yan Hsu[7], Titiksha Peetumber[4], Céline Arzel[4,8,**], Markus Öst[2,9,**]

[1]School of Environmental Sciences, University of Liverpool, Liverpool L69 3GP, United Kingdom

[2]Environmental and Marine Biology, Åbo Akademi University, 20500 Turku, Finland

[3]Centre d'Etudes Biologiques de Chizé, UMR 7372 CNRS – La Rochelle Université, 79360 Villiers-en-Bois, France

[4]Department of Biology, University of Turku, 20014 Turku, Finland

[5]Finnish Environment Institute, 00790 Helsinki, Finland

[6]Department of Biological and Environmental Sciences, University of Jyväskylä, Finland

[7]Department of Life Science, Tunghai University, Taichung, Taiwan

[8]OMPO, 59 rue Ampère, 75017 Paris, France

[9]Novia University of Applied Sciences, 10600 Ekenäs, Finland

*: these authors contributed equally and share co-first authorship

**: these authors contributed equally and share co-last authorship

Orcid IDs: BM, 0000-0001-8553-4041; FV, 0000-0001-9557-6966; KJ, 0000-0002-5568-5647; SR 0000-0001-5582-9455; OC, 0000-0002-3490-6770; CP: 0009-0004-5501-5581, BYH, 0000-0002-3799-0509; CA 0000-0002-1866-2067; MÖ, 0000-0002-2205-1437.

***Correspondence:**
Bertille Mohring
bmohring@liverpool.ac.uk

Supplementary materials S1: Linear mixed model testing for colony differences in corticosterone levels and in the magnitude of the corticosterone stress response among incubating female common eiders from two colonies (Bengtskär and Tvärminne), controlling for body mass and including a three-way interaction between colony, sample type (baseline or stress-induced) and body mass. Female identity was included as a random effect. Significant effects ($p \leq 0.05$) are presented in bold.

Dependent variable	Independent variable	χ^2	P-value	n	n_T	n_B
CORT	**Colony**	**4.745**	**0.029**	100	79	21
	**Sample type**	**407.852**	**<0.001**			
	Body mass	0.561	0.454			
	Colony x Sample type	0.015	0.901			
	Colony x Body mass	0.069	0.793			
	Sample type x Body mass	0.934	0.334			
	Colony x Sample type x Body mass	0.490	0.484			

Abbreviations: ‘n’: total sample size; ‘n_T’: sample size at Tvärminne; ‘n_B’: sample size at Bengtskär; ‘CORT’: corticosterone levels; ‘Sample type’: baseline or stress-induced level.

Supplementary materials S2: Linear models testing for colony differences in each hormone (baseline prolactin and thyroid hormones triiodothyronine and thyroxine) among incubating female common eiders from two colonies (Bengtskär and Tvärminne), controlling for body mass and including a two-way interaction between colony and body mass. Significant effects ($p \leq 0.05$) are presented in bold and nearly significant ones ($p \leq 0.10$) in bold italics.

**Dependent variable**	**Independent variable**	**F-value**	**P-value**	**n**	**n_T**	**n_B**
PRL	**Colony**	**5.839**	**0.018**	93	72	21
	Body mass	1.174	0.282			
	Colony x Body mass	1.541	0.218			
T4	Colony	0.995	0.327	31	9	22
	Body mass	0.410	0.527			
	Colony x Body mass	***3.272***	***0.082***			
T3	**Colony**	**8.922**	**0.006**	31	9	22
	Body mass	1.121	0.299			
	Colony x Body mass	0.023	0.881			

Abbreviations: 'n': total sample size; 'n_T': sample size at Tvärminne; 'n_B': sample size at Bengtskär; 'PRL': baseline prolactin levels; 'T3': triiodothyronine levels; 'T4': thyroxine levels.
